# Non-invasive biomarkers for detecting progression toward hypovolemic cardiovascular instability in a lower body negative pressure model

**DOI:** 10.1038/s41598-024-59139-8

**Published:** 2024-04-15

**Authors:** Ethan K. Murphy, Spencer R. Bertsch, Samuel B. Klein, Navid Rashedi, Yifei Sun, Michael J. Joyner, Timothy B. Curry, Christopher P. Johnson, Riley J. Regimbal, Chad C. Wiggins, Jonathon W. Senefeld, John R. A. Shepherd, Jonathan Thomas Elliott, Ryan J. Halter, Vikrant S. Vaze, Norman A. Paradis

**Affiliations:** 1 Thayer School of Engineering, Dartmouth College, Hanover, NH 03755 USA; 2 Geisel School of Medicine, Dartmouth College, Hanover, NH 03755 USA; 3 Dartmouth-Hitchcock Medical Center, Lebanon, NH 03756 USA; 4 Department of Anesthesiology and Perioperative Medicine, Mayo Clinic, Rochester, MN 55902 USA

**Keywords:** Diagnostic markers, Biomedical engineering, Blood flow, Translational research

## Abstract

Occult hemorrhages after trauma can be present insidiously, and if not detected early enough can result in patient death. This study evaluated a hemorrhage model on 18 human subjects, comparing the performance of traditional vital signs to multiple off-the-shelf non-invasive biomarkers. A validated lower body negative pressure (LBNP) model was used to induce progression towards hypovolemic cardiovascular instability. Traditional vital signs included mean arterial pressure (MAP), electrocardiography (ECG), plethysmography (Pleth), and the test systems utilized electrical impedance via commercial electrical impedance tomography (EIT) and multifrequency electrical impedance spectroscopy (EIS) devices. Absolute and relative metrics were used to evaluate the performance in addition to machine learning-based modeling. Relative EIT-based metrics measured on the thorax outperformed vital sign metrics (MAP, ECG, and Pleth) achieving an area-under-the-curve (AUC) of 0.99 (CI 0.95–1.00, 100% sensitivity, 87.5% specificity) at the smallest LBNP change (0–15 mmHg). The best vital sign metric (MAP) at this LBNP change yielded an AUC of 0.6 (CI 0.38–0.79, 100% sensitivity, 25% specificity). Out-of-sample predictive performance from machine learning models were strong, especially when combining signals from multiple technologies simultaneously. EIT, alone or in machine learning-based combination, appears promising as a technology for early detection of progression toward hemodynamic instability.

## Introduction

Hemorrhage progressing to hemodynamic instability is a leading cause of death in trauma patients^1,2^. Although many cases of hemorrhage are self-evident and recent improvements such as balanced resuscitation and advanced trauma life support (ATLS) protocols^2,3^ have improved outcomes, there remain some patients that present insidiously after trauma–occult subclinical hemorrhages. In these cases, there may be significant progression toward hemodynamic instability without any discernible changes in traditional clinical parameters^4^, including blood pressure (BP), heart rate (HR), and plethysmography (Pleth), until sudden hemodynamic deterioration requires rescue therapies. Delays in detection are associated with worse patient outcomes^5–8^.

Early detection of ongoing subclinical hemorrhage before onset of frank shock is a major unmet need. In particular, a noninvasive technology that can be applied as a wearable and act as a monitor and accurate early alarm could prevent significant morbidity and mortality. Currently, none of the existing non-invasive technologies has adequate performance with respect to underlying false-positive and false-negative rates to function in such a role. We have previously hypothesized that a multiplexed combination of non-invasive technologies that incorporates anatomic and temporal patterns via machine learning may outperform existing solutions^9^ and reported that such an approach had superior diagnostic performance^10^ in a porcine model of occult hemorrhage^11^. In particular, we observed that direct change-from-baseline impedance measurements^12^ and a multiplexed model built of absolute and impedance-change data appeared to have exceptional performances^9^.

In light of our previous results in a porcine model, we undertook evaluation of this same approach in a human model. An established method to safely mimic progression toward hemodynamic instability in humans is through use of a lower body negative pressure (LBNP) model^4,13–15^. The application of LBNP results in “a central volume shift to the lower body, which creates central hemodynamic conditions that may mimic those obtained during actual blood loss”^4^. LBNP has been used to evaluate various diagnostic signals that may assist in earlier detection of hemorrhage^8,13,16–19^. Absolute and relative vital sign and electrical impedance-based metrics are evaluated via direct analysis and via a machine-learning (ML) based approach.

## Results

### Subjects

**Figure 1:**
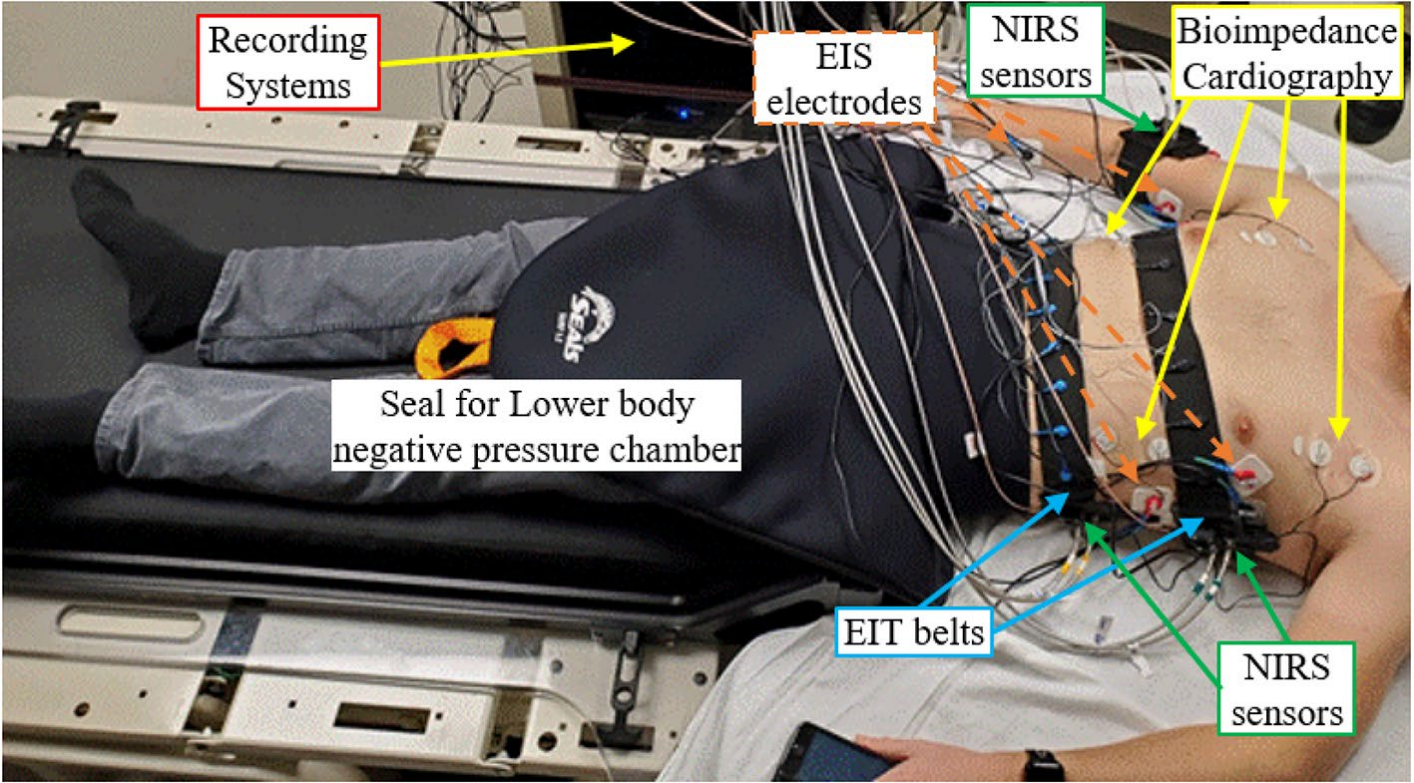
Photo of a subject with sensors attached prior to starting the LBNP experiment. The EIT belts (blue arrows) are positioned around the chest and abdomen, the EIS electrodes (orange) inject current across the thorax, abdomen, and arm, bioimpedance cardiography (BC) uses custom electrodes placed in prescribed locations above and below the heart, and 3 NIRS sensors recorded data in similar locations. Although not present in the photo (due to poor timing), an arterial line was placed on the patient’s left arm to invasively measure MAP.

Eighteen healthy male and female volunteers (11 males, 7 females) with an average (± standard deviation) age of 32.7 ± 8.2 (23–51), height of 172 ± 8.6 cm (158–193 cm), weight of 77.1 ± 10.8 kg (63–101.8 kg), and body mass index of 25.7 ± 2.6 kg/m^2^ (21.8–30 kg/m^2^) were recruited to participate in this LBNP study (January to March 2020). Figure 1 provides an overview of the measurements recorded on one subject. The study was originally powered for 25 subjects, but was stopped early because of COVID. Of the 18 subjects recruited for the study, there were impedance instrumentation issues on 2 subjects. Thus, we restricted the analysis to the 16 subjects where complete data was available. LBNP levels were increased in a step-wise manner (15 mmHg per step, held stable for 5 min per step) until a clinically evident hypotensive state developed. The average LBNP reached over all subjects was 80.0 ± 11.7 mmHg (60–100 mmHg), with 2 subjects stopping at 60 mmHg and one subject continuing to 100 mmHg.

**Figure 2:**
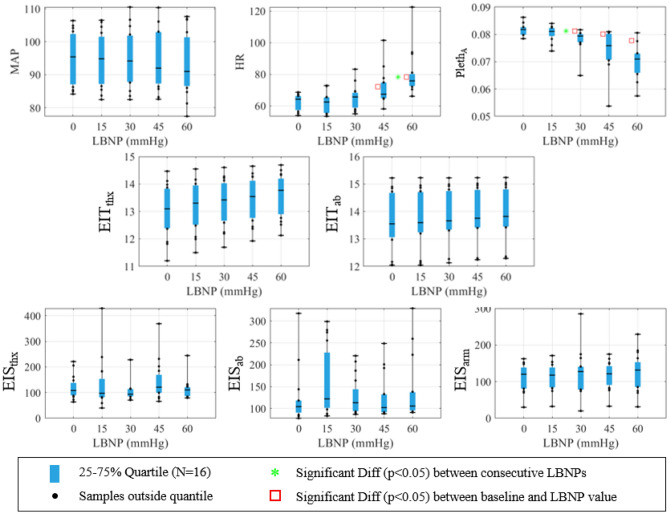
Absolute metric values displayed via quartile box-and-whisker plots. Black dots indicate any subject outside of the quartiles and the median value is shown as a black line segment. Red squares indicate significant differences between the given LBNP level and baseline.

**Figure 3:**
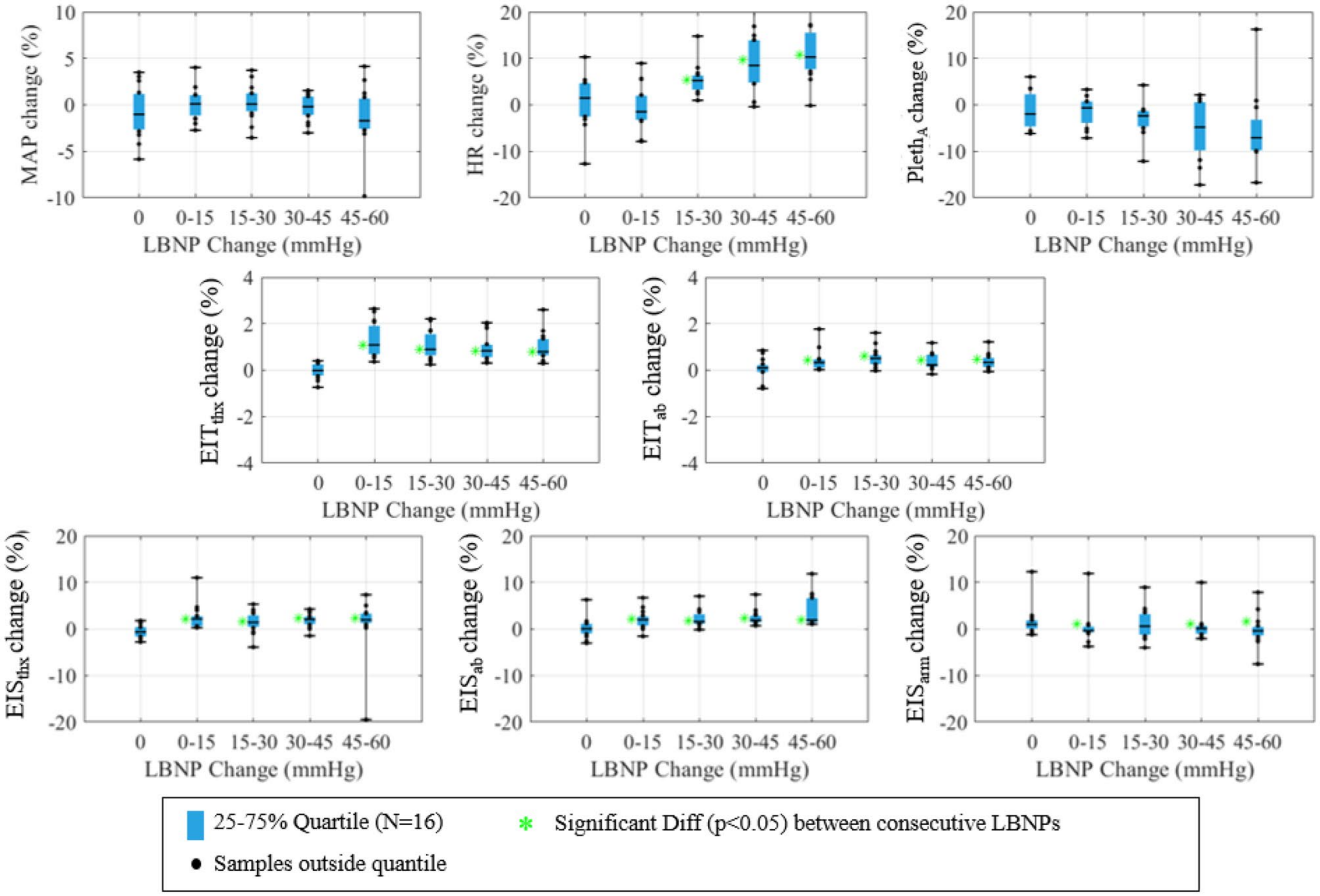
Relative metric value displayed via quartile box-and-whisker plots. Black dots indicate any subject outside of the quartiles and the median value is shown as a black line segment. Green stars indicate significant differences between consecutive LBNP levels.

**Figure 4:**
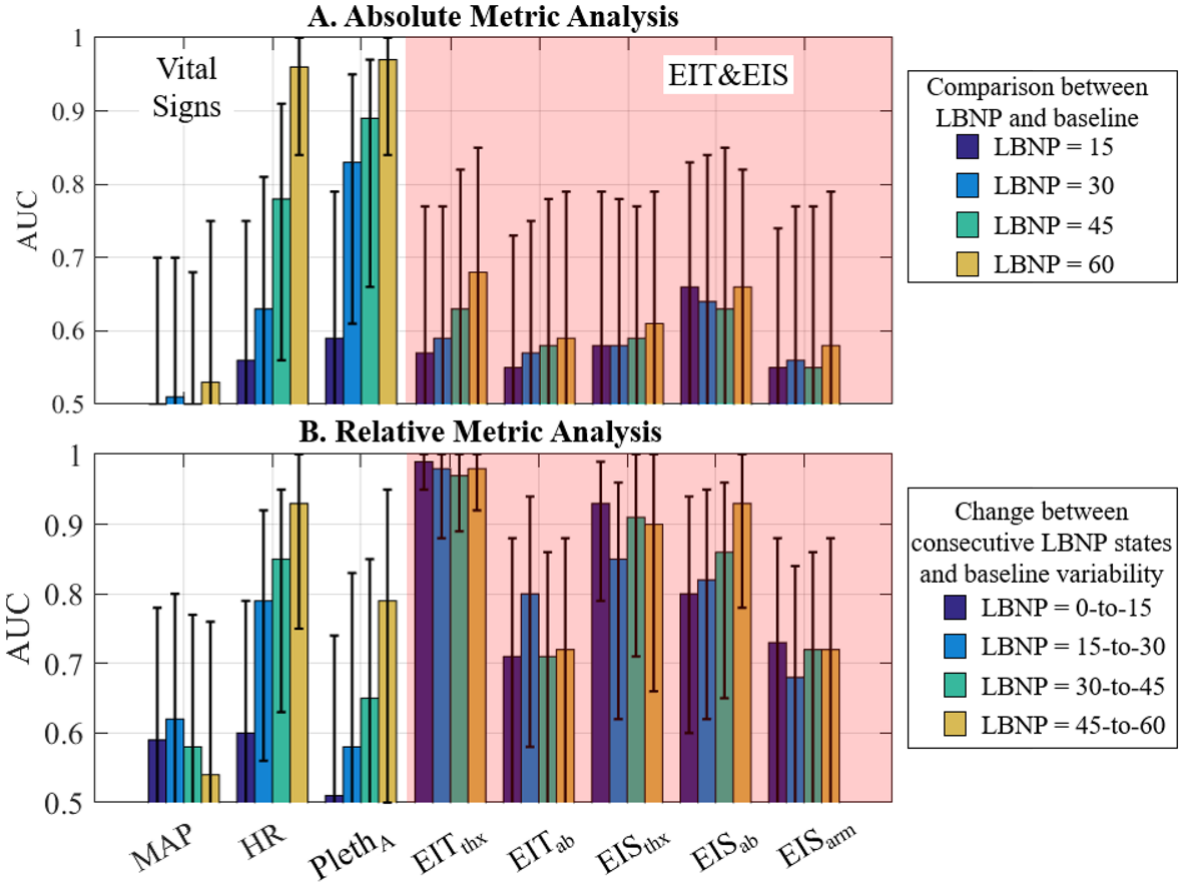
AUCs (bars) and 95% confidence intervals (whiskers) of the (**A**) absolute and (**B**) relative analysis for the vital signs and EIT/EIS metrics at each LBNP level (**A**) or each LBNP change (**B**). All analyses compare metric values (at the specific LBNP level) or change in metric values (over the specified LBNP change) to baseline or baseline variability, respectively—i.e. these are bleed versus no-bleed analyses performed at different LBNP levels. No comparisons are performed between the different LBNP levels, e.g. comparisons between metric changes from LBNPs of 0-to-15 to those from LBNPs of 15-to-30 are not considered.

The absolute and relative data are displayed as box-and-whisker plots (25–75% with N = 16 samples for each) in Figs. 2 and 3. Note that Pleth_A_ is a metric calculated from the Pleth waveform (see “Methods” section) and EIT and EIS metrics are average, filtered impedance data corresponding to the noted sites (thx = thorax, ab = abdomen). Minute-based averages of all the data is additionally shown in Supplementary Appendix A.1. For the absolute metrics, a red square indicates significant differences between the current LBNP level and baseline (p < 0.05), and a green star indicates significant differences between consecutive LBNP levels (p < 0.05). The relative metrics in Fig. 3 show the change in metric values over the 5-min baseline period (0 mmHg) and across consecutive LBNP levels (0-to-15, 15-to-30, 30-to-45, and 45-to-60 mmHg). A description of the baseline-change calculation is provided in Supplementary Appendix A.2. Green stars in these plots indicate a significant difference between the metric changes across consecutive LBNP levels and baseline variability (p < 0.05). Bar graphs of area-under-the-curves (AUCs) are presented (Fig. 4A,B) for the absolute and relative metrics with overlaid 95% confidence intervals (CI), where AUCs are calculated from the receiver operating characteristic (ROC) curves.

### Absolute metric analysis

The absolute metrics and associated AUCs are presented in Figs. 2 and 4A. HR and Pleth_A_ trends appear to track with increasing LBNP, while only subtle trends appear in the remaining metrics (Fig. 2). However, there is significant overlap in the distributions between LBNP levels indicating a limited ability to differentiate states. Only HR and Pleth_A_ had significant differences; (1) with respect to baseline, starting at LBNP levels ranging from 30 mmHg (Pleth_A_) to 45 mmHg (HR)—indicated by red squares, and (2) between consecutive LNBP levels from 45-to-60 for HR and 15-to-30 for Pleth_A_—indicated by green starts. The best AUCs were 0.66 (EIS_ab_), 0.83 (Pleth_A_), 0.89 (Pleth_A_), and 0.97 (Pleth_A_) for LBNP levels of 15, 30, 45, and 60 mmHg, respectively, where CIs are only above 0.8 for HR and Pleth_A_ for an LBNP of 60 mmHg. Excluding HR and Pleth_A_, most of the AUCs in Fig. 4A are close to 0.5 (all are under 0.7), indicating a limited ability to differentiate states. The best AUC across impedance metrics is 0.68 (EIT_thx_) with a CI of (0.50–0.84) at an LBNP level of 60 mmHg. The EIS data shown here correspond to the impedance magnitude and frequency that yields the maximum AUC. Specifically, the best frequencies were all less than or equal to 560 Hz, except for the comparison of 30 mmHg to baseline for the thorax (26.5 kHz). One could confidently say that hypovolemia induced via the LBNP model remains subclinical to vital sign metrics for an LBNP of 15 mmHg and perhaps up to 30 mmHg, although Pleth_A_ shows significant differences with an AUC of 0.83 (CI 0.59–0.96).

### Relative metric analysis

**Figure 5:**
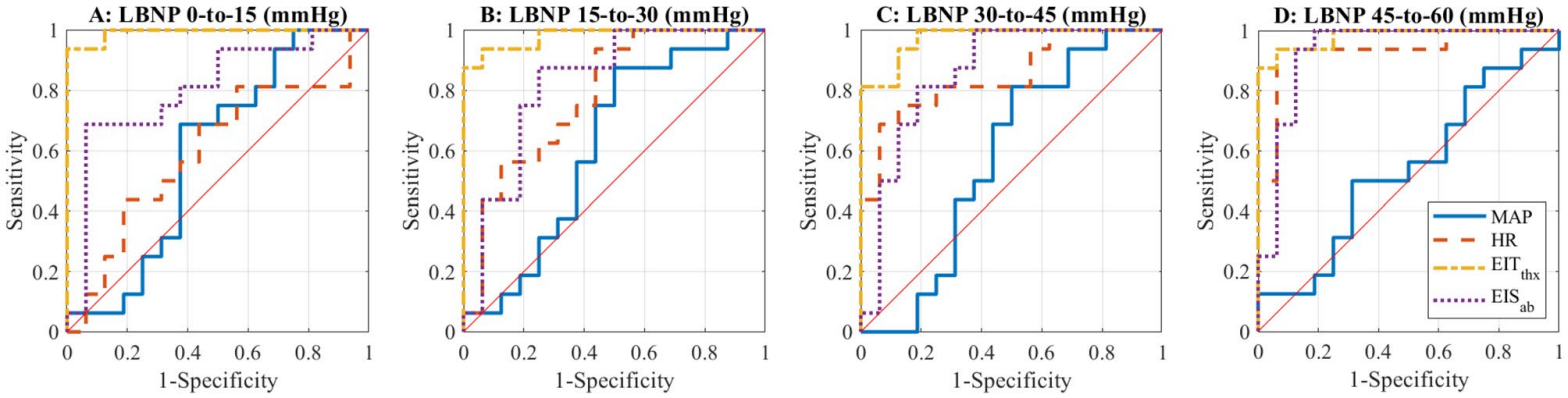
(**A–D**) ROC curves corresponding to MAP, HR, and the best metrics from EIT (thorax) and EIS (abdomen) for the relative analysis of change between consecutive LBNP levels and baseline change.

The relative metrics and associated AUCs are presented in Figs. 3 and 4B. Overall, the relative metrics appear very good at differentiating between increasing levels of hypovolemia (i.e., change between consecutive LBNP levels), with HR and EIT/EIS measurements recorded from the thorax and abdomen showing significant differences (green stars in Fig. 3). No metric has perfect separation from the baseline variability, but the EIT thorax data, EIT_thx_, comes closest, achieving AUCs of 0.99, 0.98, 0.97, and 0.97 for each LBNP level studied with CIs above 0.92 for LBNP changes of 0-to-15 mmHg and 45-to-60 mmHg. The EIT_thx_ metric outperforms all other metrics (Fig. 4B) with a high degree of efficacy in identifying hypovolemia (AUC = 0.97, CI 0.95–1.00) across all levels of LBNP explored here. Some other techniques achieved AUCs > 0.9 for a subset of LBNP ranges: HR for LBNP change of 45-to-60 mmHg (0.93, CI 0.75–1.00), EIS on the thorax for LBNP changes of 0-to-15 (0.93, CI 0.79–0.99) and 45-to-60 mmHg (0.91, CI 0.71–1.00), and EIS on the abdomen for LBNP change of 45-to-60 mmHg (0.93, CI 0.78–1.00). The EIS data presented here correspond to the impedance magnitude and frequency that yields the maximum AUC. In general, these optimum frequencies fell between 8 and 27 kHz. Although the impedance changes are very consistent, they are small. For example, the median percent increases in EIT_thx_ are 1.09%, 0.89%, 0.83%, and 0.79% with minimum increases of 0.36%, 0.24%, 0.31%, and 0.29% for LBNP changes from 0-to-15, 15-to-30, 30-to-45, and 45-to-60 mmHg, respectively. ROC curves (Fig. 5A–D) from MAP, HR, the best EIT metric (EIT_thx_), and best EIS metric (EIS_ab_) illustrate the superior performance of EIT_thx_ compared to other metrics. When requiring 100% sensitivity at the smallest LBNP change (0-to-15 mmHg), EIT_thx_ has a much higher specificity of 87.5% compared to the best vital sign specificity of 25% (MAP).

### ML analysis: individual technologies

**Figure 6:**
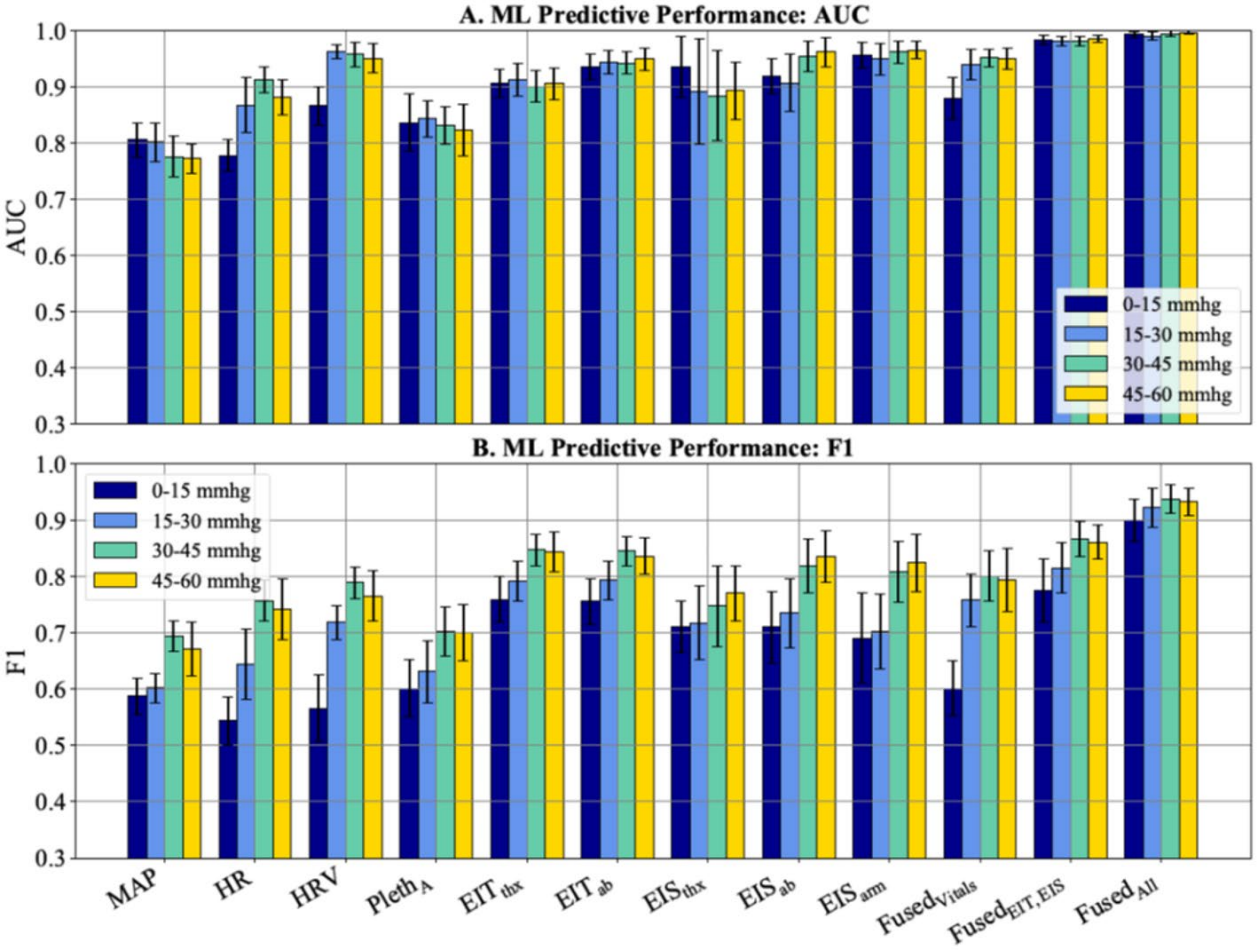
Out-of-sample random forest performance results (bars) and 95% confidence intervals for each 15-fold cross validation (whiskers) for models trained on 7-min slopes from vital signs, impedance, and fused metrics, shown for each LBNP change. Fused results represent performance of majority voting ensembles that combine vital sign metrics (Fused_Vitals_), impedance metrics (Fused_EIT,EIS_), and all metrics (Fused_All_). All analyses compare slopes over the specified LBNP change to baseline—i.e. they perform bleed versus no-bleed analyses. No comparisons are performed between different LBNP levels, e.g. comparing slopes from LBNPs of 0-to-15 to those from LBNPs of 15-to-30 are not considered.

The ML approach relied on a binary time-series classification (TSC) method using a random forest model, 15-fold cross-validation, and its overall performance was assessed using out-of-sample diagnostic performance. The out-of-sample AUC and F1 performance results for the binary TSC models using 7-min slope windows filtered for each LBNP change are shown in Fig. 6. F1 scores were used in addition to AUC to accurately measure the predictive performance of the ML models; unlike the balanced data set used in the above absolute and relative metric analyses, the ML data set often contained imbalanced data with more hypovolemic samples than normovolemic samples, making F1 score a more apt performance metric^20^. Individual and fused results are shown for models trained on 7-min slopes because that window length yielded the best out-of-sample results (see Supplementary Appendix A.3 for performance of other time windows). The mean vital sign F1 scores for an LBNP change of 0-to-15 mmHg are 0.59, 0.54, 0.57, and 0.60 for MAP, HR, HRV, and Pleth_A_, respectively. The EIT and EIS technologies performed generally better than vital signs, yielding mean F1 scores of 0.76, 0.76, 0.71, 0.71, and 0.69 for EIT_thx_, EIT_ab_, EIS_thx_, EIS_ab_, and EIS_arm_, respectively.

### ML analysis: multi-technology

In addition to measuring the out-of-sample predictive performance of each individual technology, each model’s predictions were combined and used in a majority voting scheme to measure the predictive performance of combined technologies. Combining traditional vital sign technologies (Fused_Vitals_) yielded F1 scores of 0.60, 0.76, 0.80 and 0.79 for the 0-to-15 mmHg, 15-to-30 mmHg, 30-to-45 mmHg, and 45-to-60 mmHg LBNP changes, respectively. Combining impedance-based technologies (Fused_EIT,EIS_) yielded F1 scores of 0.78, 0.82, 0.87 and 0.86 for each LBNP change respectively. Combining all non-invasive technologies (Fused_All_) yielded mean F1 scores of 0.90, 0.92, 0.94, and 0.93 for each LBNP change respectively, far outperforming all individual technologies and other multi-technology models (see last 3 sets of results in Fig. 6).

## Discussion

We have previously described the high performance of non-invasive impedance-based measurements in the early identification of occult hemorrhage in a porcine model^12^ and the ability of machine learning to improve this performance through the application of multivariable algorithms^9^. We have now extended this observation into an established human model of LBNP progressive circulatory instability from central hypovolemia. The present investigation evaluated the performance of multiple technologies to detect early subclinical changes in LBNP in an absolute and relative (change) sense compared to standard vital sign metrics.

Impedance-based non-invasive sensing performed best when used as relative metrics. The performance of relative EIT_thx_ is worth noting. It had an AUC of 0.99 (CI 0.95–1.00) at the smallest LBNP change (0-to-15 mmHg) and when requiring 100% sensitivity it yielded an extremely high specificity of 87.5% especially when compared to the 25% specificity achieved by the best vital sign metric (MAP) at this LBNP change. LBNP 0-to-15 mmHg is almost always clinically occult and existing detection solutions perform poorly at this LBNP level with high rates of false negatives. Given these encouraging results, it is important to highlight the clinical value of analyzing *relative metrics*. Based on Ref.^14^, LBNP levels of 15, 30, 45, and 60 mmHg respectively correspond to blood losses of 93, 193, 313, and 451 ml, and consequently, for LBNP levels *changes* of 0-to-15, 15-to-30, 30-to-45, and 45-to-60 mmHg there should be a corresponding increased blood volume loss of 93, 100, 120, and 138 ml. Thus, the relative metric-based analysis essentially evaluates if one can detect an increase in blood loss relative to no blood loss for these particular volumes. The data here suggests that a simple threshold applied to the change in EIT_thx_ is sufficient to detect these small occult-level blood volume changes (note that EIT_thx_ change data are all above zero in Fig. 3), i.e. no apparent baseline (healthy state) is needed in the relative metric-based analysis. At the same time, Fig. 3 shows that the change in the EITthx metric cannot determine the severity of the bleed, i.e. all changes evaluated (0-to-15 up to 45-to-60) appear essentially the same. Although not considered here, it would be interesting, in a larger study, to investigate if any combination of absolute or relative metrics using ML techniques could help determine the severity of the bleeds in terms of cumulative volume or rate.

Similar to previous investigations^12^, we found that traditional vital signs measured with standard monitors such as ECG and plethysmography performed poorly in early detection of LBNP progression. EIT outperformed these metrics, with an observed AUC of 0.99 (CI 0.95–1.00) for EIT_thx_, while the best clinical metric at the same LBNP level exhibited an AUC of only 0.60 (CI 0.38–0.79) (HR).

As in our earlier porcine study, impedance performs poorly as an absolute metric (e.g., see Table 3 in Ref.^12^). This can largely be attributed to the variation in body size and habitus between study participants. Given similar tissue impedance characteristics of all subjects (e.g., assuming muscle or adipose impedance is similar between subjects), a large subject will have a larger impedance compared to a small subject due to variations in electrode spacing and underlying constituent tissues. Unfortunately, simple calibration factors are generally not adequate, as the impedance is dependent on not only the subject’s size (cross-sectional area), but also tissue composition (e.g., percent adipose tissue)^21^, hydration status (electrolyte content)^22^, and recent activity levels^23^. ROC analysis of EIT metrics including various height, weight, body mass index (BMI), and body surface area (BSA) factors were evaluated; no factor explored yielded notable improvements in the absolute analysis.

With respect to out-of-sample predictive performance, a clear contrast emerges between the strong out-of-sample performance achieved using data generated by the impedance-based technologies as compared to the other technologies. Only impedance-based technologies were able to achieve AUCs > 90% across all four consecutive LBNP levels (see Fig. 6A). Mean AUC and F1 scores for impedance-based technologies dominated scores from all other individual technologies for the 0-to-15 mmHg LBNP change. HRV models tended to prioritize specificity over precision (i.e., do not flag subjects as hypovolemic unless they are very certain), resulting in strong AUCs, but weaker F1 scores.

Pleth-based results from Convertino and others, e.g.^8,18,19^, appear promising but may have limitations when applied broadly. For example, investigators have established that in LBNP studies their Pleth-based metric can be separated into subjects that have high and low tolerance to hypovolemia^15,17^. This may impair detection of blood loss in “high tolerance” individuals. As we hypothesize that impedance is a measure of the sensitivity to the blood loss and not to the body’s *response* to the blood loss, our impedance-based technology might not be susceptible to false-negative findings in subgroups of subjects.

The near perfect performance of EIT for the initial 0–15 mmHg step is particularly encouraging. This early period in the transfer of circulating blood volume is generally not detectable clinically and or with alternative existing technologies (Fig. 5A). A clinical alarm that activated just after initial hemorrhage would give clinicians the largest possible window for evaluation and treatment before onset of frank deterioration.

A limitation of the study was the small sample size. A larger study could have allowed for additional sub-analysis investigating dependencies on factors such as age, sex, height, and BMI. In terms of non-impedance related metrics, other studies have performed these types of analyses, e.g. see^24^ for a review. If the performance described here is replicated in larger, future studies, development of a clinical early detection alarm system seems possible as these results indicate that low rates of false negative and false positive may be possible even in heterogenous clinical populations. A future wearable system may be reduced to a belt of electrodes and a small wireless device.

There are a number of limitations to the results reported here. While LBNP may be among the best available mimics for early hemorrhage it has all the limitations of a model system, and performance in actual clinical setting may be inferior. LBNP subjects in this study are all healthy volunteers. Patients will have significant phenotypic heterogeneity, may be taking medications that alter physiologic responses, and will likely be receiving intravenous fluids at variable rates^25^. A shortcoming of the ML-based approach is the reliance on calculating slopes of metrics from an overall LBNP-step-based experiment (5 min at each level). The slope calculations allowed for a large-enough database for ML training, but do not quite match the stepped, physical scenario of the LBNP model. However, the slope-based approach is how we envision the technology being implemented in practice. One additional limitation associated with the length of time-windows used for electrical impedance-based metric calculations should be noted. While the performance observed is exceptional using long time-windows (5-min here and 1-min in Ref.^12^), for shorter duration windows, one might expect respiratory and cardiac-related signals to reduce the performance as these signals will no longer be removed through the averaging operation. Window length should continue to be considered as the technology is further developed.

## Conclusions

Impedance-based noninvasive technologies appear to have promising diagnostic performance in the early detection of hypovolemia in a LBNP model. This is especially true when evaluated as a change with respect to baseline, and when incorporated in a multivariate machine learning algorithm.

## Methods

A previously described step protocol for LBNP^14^ was used to model hypovolemic cardiovascular instability in a cohort of healthy subjects. Prior to the study day, all subjects provided written informed consent after all procedures and risks of the study were fully explained, and the study was approved by Mayo Clinic’s Institutional Review Board. All human research was performed in accordance with relevant guidelines/regulations. Subjects were continuously monitored during the experiment via observation and vital signs to ensure safety.

### Experimental design

Subjects were instrumented with ECG and plethysmography for non-invasive vital sign monitoring, and a brachial arterial line was invasively introduced to capture mean arterial pressure (MAP). Non-invasive *test* devices included electrical impedance tomography (EIT) (SenTec AG, Landquart, Switzerland), electrical impedance spectroscopy (EIS) (Sciospec Scientific Instruments GmbH, Germany), Near infrared spectroscopy (NIRS) (custom-device^9^), and bioimpedance cardiography (BC) (Starling, Baxter, USA) as shown in Fig. 1. Only vital sign metrics and EIT/EIS data are presented in this report. High subject-to-subject variability with the particular NIRS configuration and its coupling to the subject skin limited its value, and BC did not provide additional value relative to past reports of its performance in LBNP studies (see Supplementary Appendix A.4)^13^. The impedance spectroscopy device (EIS) recorded electrical impedance spectra from 3 sites (chest, abdomen, and arm), and the impedance tomography device (EIT) collected multiple impedance measurements from two 16-electrode belts positioned around the subjects’ chest and abdomen. The belt was placed just below the nipple line (see Fig. 1), so belt placement was essentially the same across sexes. The pressure waveforms/values, ECG voltages, Pleth voltages, and LBNP levels were captured using LabChart 7.0 (ADI, Sydney, Australia).

Prior to LBNP progression each subject rested in the supine position for approximately 5 min. The LBNP levels proceeded as follows: baseline, 15 mmHg, 30 mmHg, 45 mmHg, 60 mmHg, 70 mmHg, 80 mmHg, 90 mmHg, and 100 mmHg. The experiment aimed to hold the negative pressure stable for 5 min at each level. There was a necessary transition period, which occurred *within* each 5-min interval. Consequently, the average stable period of each level was 4.2 min, i.e. ~ 48 s transition periods. At each 5-min time-point the LBNP moved to the next level. Each subject underwent an LBNP progression from a baseline hemodynamic state until a clinically evident hypotensive state developed. The target hypotensive state was achieved when any of the following criteria was met: (a) systolic BP of $\le$ 70 mmHg or (b) the subject asked to stop because of symptoms typically including, but not limited to, lightheadedness or nausea.

### Data

The collected data was averaged during the stable periods of each LBNP level. Only the data associated with LBNP levels of 0, 15, 30, 45, and 60 mmHg were considered—to focus on clinically occult states. The different LBNP levels are referred to as *hypovolemia states* as they are meant to mimic a volume contraction secondary to processes such as hemorrhage.

Baseline data was collected for each subject for five minutes before the LBNP process was initiated (LBNP = 0 mmHg), and the protocol took between 28 and 54 min. The beat-to-beat HR was calculated from R-peaks extracted from the ECG data. Because only a single channel of Pleth data was available for some subjects, a standard blood oxygen calculation was not possible. As a surrogate, the trough-to-trough area-under-the-curve was computed for each Pleth time-series (similar to that used in Ref.^12^) and denoted as *Pleth*_*A*_.

The EIS data recorded the complex impedance (real and imaginary or magnitude and phase) at 100 logarithmically-spaced frequencies ranging from 100 Hz to 1 MHz at a rate of ~ 1 spectra per minute at three anatomical locations. Results are presented for the impedance magnitude and frequency yielding the maximum AUC at each site considered. We denote the impedance spectroscopy results as EIS_thx_, EIS_ab_, and EIS_arm_ for the thorax, abdomen, and arm sites, respectively. There is an abundance of EIT data, which could be used in a standard way to image cross-sections of the thorax and abdomen. However, here we average a subset of impedance data (specified in Supplementary Appendix A.5) at each time and perform checks to ensure that the data is valid (i.e., electrode–skin contact is good), similar to that described in Ref.^12^. This approach yields a single belt location-specific mean impedance value ± (standard deviation) for each LBNP level. We denote the impedance tomography results as EIT_thx_ and EIT_ab_ for the thorax and abdomen sites, respectively.

### Analysis

The data is visualized in terms of 25–75 quartile box-and-whisker plots for each metric and LBNP level. Each box-and-whisker plot is produced using one metric value from each subject. Two main types of analyses were performed: absolute and relative. The *absolute analysis* explored the potential of using the raw values of each metric at a specific LBNP level to distinguish between normovolemia (LBNP = 0 mmHg) and hypovolemia (LBNP > 0 mmHg) states. The *relative analysis* investigated how significantly different a metric change between two LBNP levels (e.g., a metric difference between LBNP levels of 0 and 15 mmHg) was with respect to baseline variability (metric change over the 5-min of baseline). As not all metrics values yielded normal distributions, Wilcoxon rank sum tests were used with significance defined for p-values < 0.05. Additionally, receiver operating characteristic (ROC) analysis was performed on both absolute and relative metrics and the area-under-the ROC curves (AUCs) are reported^26^; confidence intervals of the AUCs are also computed using 500 bootstrap samples.

### ML and analysis

A binary time series classification (TSC) method was employed to assess the out-of-sample diagnostic performance of (1) each individual technology and (2) the combination of all technologies for identifying hypovolemic states. A random forest model was used to classify subjects as either hypovolemic or normovolemic using 15-fold cross validation in which data from 13 randomly chosen subjects were used for training and the remaining 3 for testing. The random forest models; did not set a max tree depth (as specifying the tree depth can result in underfitting^27^), 500 estimators (trees) were used because that number represents a typical default value^28^, and splitting criteria was based on the Gini index, which are appropriate for classification problems^29^. Similar to the absolute analysis, the ML approach was designed to determine whether or not subjects were in a normovolemic (LBNP = 0 mmHg) or hypovolemic state (LBNP > 0 mmHg). Data produced from each technology was initially modeled individually; each model’s predictions were then used in a majority voting scheme to evaluate any performance improvements associated with the multi-modal signals captured. A majority voting ensemble was employed as this approach often exceeds performance of a single machine learning model in clinical settings^30–32^. Combinations considered for ML ensembles included vital metrics (Fused_Vitals_), impedance metrics (Fused_EIT,EIS_), and all metrics (Fused_All_). Instead of using the entire training set for each ML model in the ensembles, we use each technology’s data individually, and then perform the majority vote using predictions made by each technology’s random forest model. This simplified majority voting method is easier to interpret because it separates performance achieved by each technology, and yet achieves strong combined performance.

In order to apply supervised learning models to the time series data for each technology, deterministic features were generated by calculating the slope across rolling time windows of increasing length. Slopes generated during the normovolemic and hypovolemic states were treated as class 0 and class 1, respectively. Due to the limited normovolemic data produced during the study, the normovolemia dataset was created by concatenating the slopes across the 2-, 3-, and 4-min rolling time windows. This is a valid approach to data augmentation given that the subject’s baseline values were observed to be stable across all technologies. Each random forest model was trained and tested using 111 normovolemic samples and between 257 and 284 hypovolemic samples depending on the window length used to calculate hypovolemic slopes, representing a range of binary class imbalances of 43.2% to 39.1%. The number of features used for each technology along with a list of feature names are provided in Supplementary Appendix A.6 (their corresponding importance are noted in Supplementary Appendix A.7). F1 scores were used in addition to AUC to accurately measure the predictive performance of the ML models; unlike the balanced data set used in the absolute and relative metric analyses, the ML data set often contained imbalanced data with more hypovolemic samples than normovolemic samples, making F1 score a more apt performance metric^20^. Additional features were generated for some technologies; for example, raw, millisecond-level HR data was used to generate 20 individual minute-level heart rate variability (HRV) features from the time, frequency, and nonlinear domains using the PyHRV library (Ref.^33^ and/or see Supplementary Appendix A.8 for the list of features). Many modern TSC applications can be effectively approached through the use of deep learning^34^, but the limited size of the dataset meant that relying on deep learning models for TSC in this instance was not feasible.

### Supplementary Information

Supplementary Information.

## Data Availability

The data that support the findings of this study have been made into an open-source data set and are available at 10.5281/zenodo.10119427.
