# Supplementary material for: Non-invasive biomarkers for detecting progression toward hypovolemic cardiovascular instability in a lower body negative pressure model
Source: Sci Rep. 2024 Apr 15;14:8719. doi: 10.1038/s41598-024-59139-8 (PMC11018605; doi:10.1038/s41598-024-59139-8)
Supplement: Supplementary file 1 — Supplementary Information. [file 41598_2024_59139_MOESM1_ESM.docx]

**Appendix: Non-Invasive Biomarkers for Detecting Progression Toward Hypovolemic Cardiovascular Instability In A Lower Body Negative Pressure Model**

**A.1. Minute-based Average Data**

The minute-based average data for the vital signs (MAP, HR, and Pleth_A_), EIT data (EIT_thx_ and EIT_ab_), and EIS data (EIS_thx_, EIS_ab_, and EIS_arm_) are given in Fig. A.1. The baseline period corresponds to the -5 to 0 minutes interval. LBNP levels are increased at the beginning of each 5-minute intervals (i.e., 0-5, 5-10, 10-15, and 15-20 minutes) with associated LBNP levels of 15, 30, 45, and 60 mmHg, respectively. Each curve corresponds to an individual subject. The vital signs are plotted in an absolute sense because the trends are easily discerned when viewed in this way. The electrical impedance data is plotted in a relative sense (i.e., as a change from baseline) because the trends are very hard to discern given the moderately large subject-to-subject variability in baseline values. These plots are supplied to give the reader a richer understanding of the data collected for the study.

**
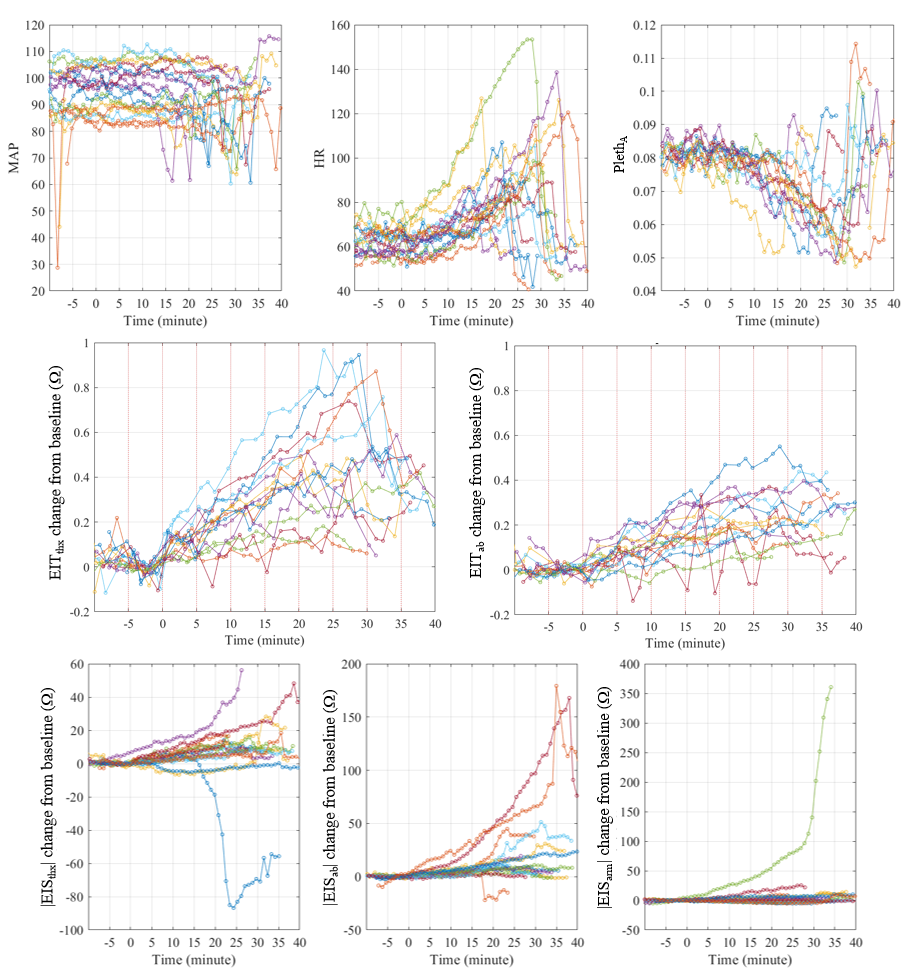
**

**Figure A.1. Minute-based average values of the vital signs (MAP, HR, and PlethA), EIT data (EIT_thx_ and EIT_ab_), and EIS data (EIS_thx_, EIS_ab_, and EIS_arm_). The baseline period corresponds to the -5 to 0 minutes interval. LBNP levels are increased at the beginning of each 5-minute intervals (i.e., 0-5, 5-10, 10-15, and 15-20 minutes) with associated LBNP levels of 15, 30, 45, and 60 mmHg, respectively. Each curve corresponds to an individual subject.**

**A.2. Baseline Description**

The baseline variability for each metric was calculated as the average change over the 5-minute baseline period. Specifically, a 1^st^ order linear model, $y_{fit}=mt+b$, was fit to the data (see Fig. A.2 for EIT data examples). The average change over the 5-minute period was then calculated as $5m/(5m+b)\times100$%. The EIT data is interesting to illustrate because of the gaps in its timeline. There was electromagnetic interference between the EIT and bioimpedance cardiography (BC) systems requiring data collection to be temporally interleaved between the two systems (with a switching interval of 3 minutes). While this reduced the total amount of data recorded by each individual system (as can be seen in Fig. A.2), the high frame rate acquisition provided sufficient data for model fitting.

**
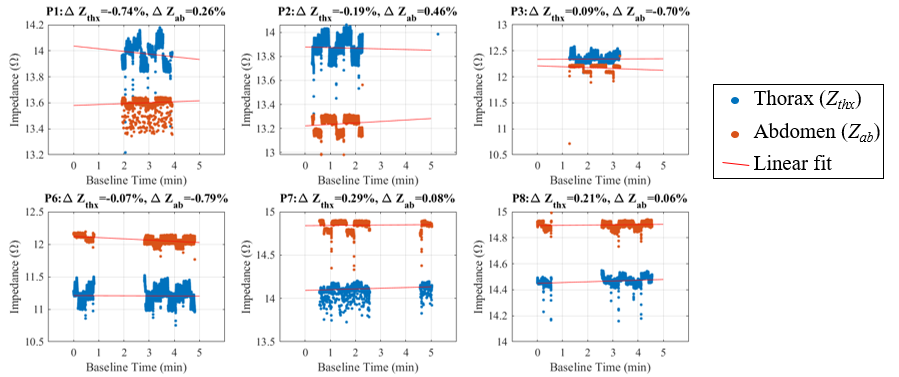
**

**Figure A.2. Six examples of calculations of the baseline variability for the EIT metrics. The blue and orange dots represent the average impedance at every frame of recorded data and the red lines are the linear best fits to the data. The titles in each figure shows the subject number and the thorax and abdomen relative change.**

**A.3. ML Results using Multiple Time-Windows for Slope Calculations**

This section shows ML results in terms of AUC (Fig. A.3.A-C) and F1-scores (Fig. A.4 A-C) for out-of-sample testing using rolling time windows for slope calculations of 2-3 minutes (small), 4-5 minutes (medium), and 6-7 minutes (large). Results in the manuscript exclusively report results using 7-minute time windows. The time-window length was an important parameter for analyzing the ML results, as one can see by the vastly different performance using 2-3 minutes versus 6-7-minute time windows. The performance as a function of time-window length is largely due to experimental design. This is because of the 5-minute time windows at which the LBNP was held constant at each level. Thus, if slopes are calculated over 2-3 minute rolling time windows, then some slopes represent changes over a fixed LBNP level. In these cases, data generated from all technologies should be stable, so slopes calculated would not capture any usable signal for time series classification. In contrast, 6-7 minute time-window-based slopes guarantee that there is a change in LBNP level and presumably the slope captures this LBNP change information. In the planned use of this technology subjects would be actively bleeding. So, a rolling time-window based analysis will likely be revisited in future work as this technology is further developed.

**
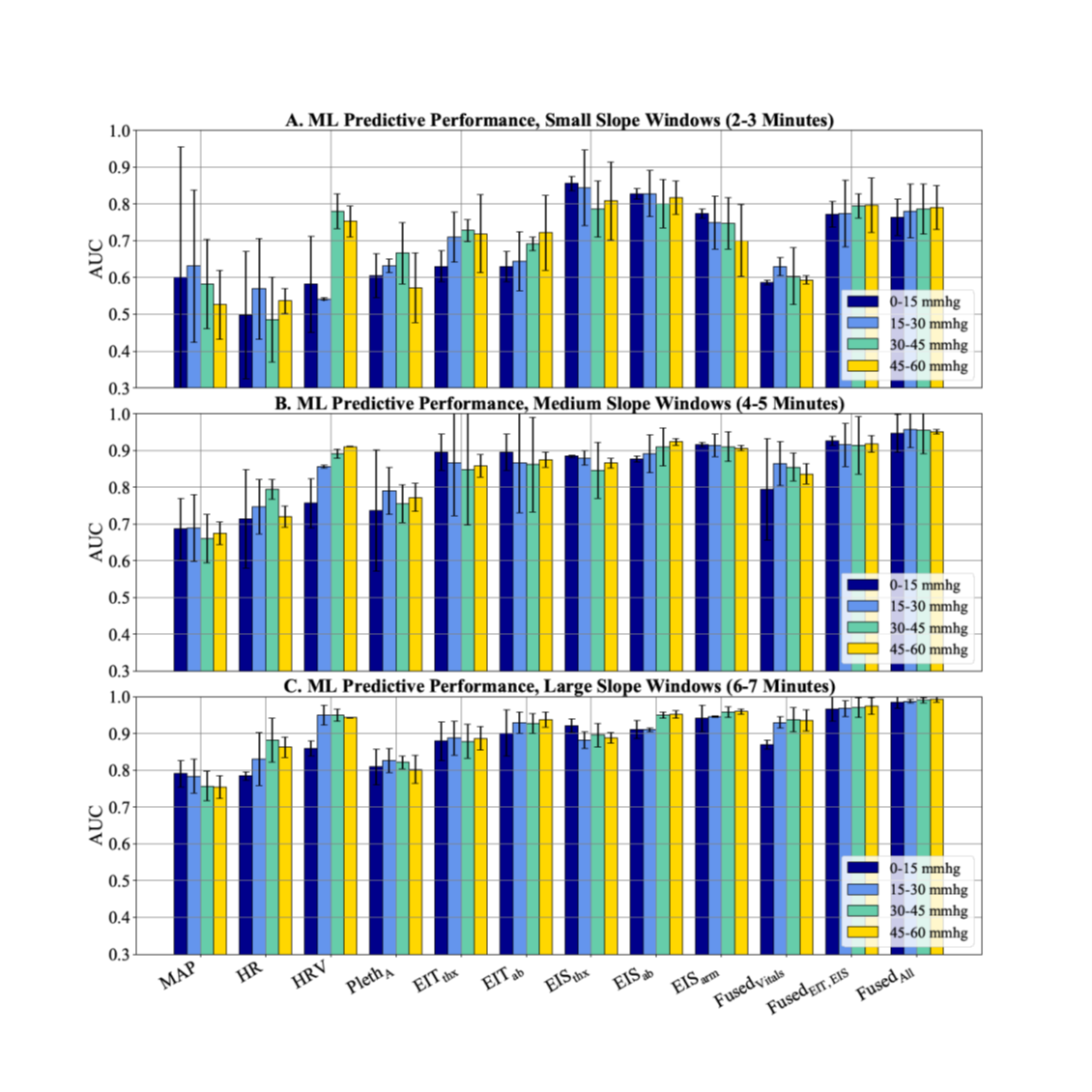
**

**Figure A.3. ML results in terms of AUCs (A-C) for out-of-sample testing using time windows for slope calculations of 2-3, 4-5, and 6-7 minutes. Results in the manuscript exclusively report results using 7-minute time windows.**

**A.4. Absolute and Relative Bioimpedance Cardiography Metrics**

As noted in the manuscript the BC metrics did not provide additional value relative to past reports. The cardiac output (CO) absolute metric results (Fig. A.5) are very similar to those reported in Fig. 2 from [13], except error bars in [13] were based on standard error whereas ‘error’ bars here (Fig. A.5) are given by 25-75% quartiles – providing a better illustration of the distribution of the data. Additional metrics reported from the BC system and shown here are the stroke volume (SV), stroke volume variability (SVV), and the ‘total fluid content’ (TFC). The TFC is actually one over the BC measured impedance, and consequently is a very similar metric to the *EIT_thx_* and *EIS_thx_*. The resulting AUCs of the absolute and relative metrics are shown in Figs. A.6.A-B. The CO and SV perform best for the absolute analysis achieving similar performance to *HR* and *Pleth_A_* vital signs (see Fig. 4). The best performance of the BC, in terms of detecting subclinical hemorrhage (LBNP < 30 mmHg), is in the relative analysis where the CO achieved an AUC of 0.8 and 0.87 for 0-to-15 and 15-to-30 LBNP levels, respectively. These results are not as good as the EIT_thx_ or EIS_thx_ (see Fig. 4), and the extent of the confidence interval (whiskers) is very wide for CO, which further reduces enthusiasm for the BC technology in this application.

**
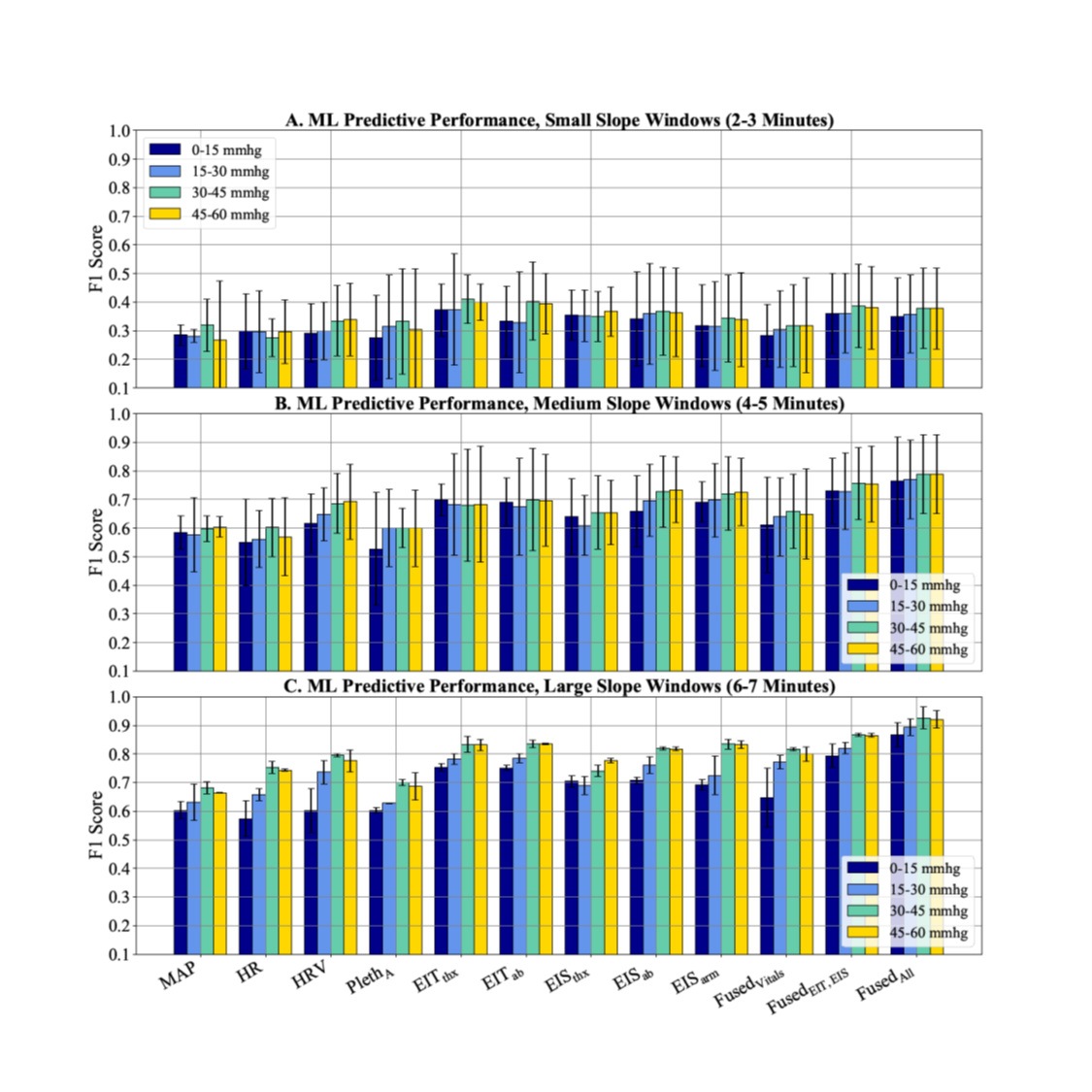
**

**Figure A.4. ML results in terms of F1 scores (A-C) for out-of-sample testing using time windows for slope calculations of 2-3, 4-5, and 6-7 minutes. Results in the manuscript exclusively report results using 7-minute time windows.**


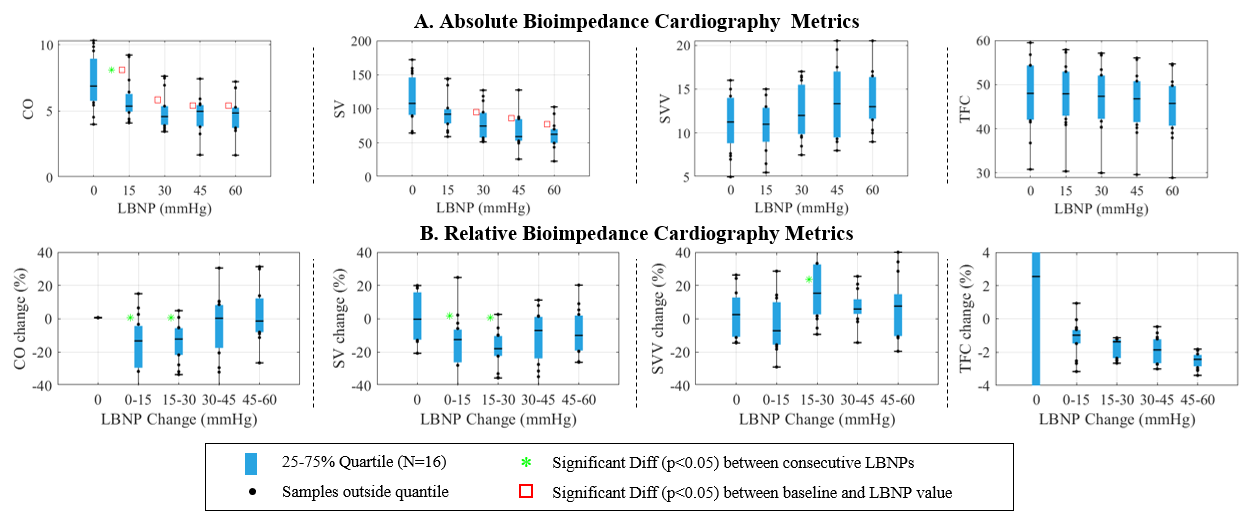


**Figure A.5. Absolute (A) and relative (B) metrics output from the** b**ioimpedance cardiography device. The metrics shown are the cardiac output (CO), stroke volume (SV), stroke volume variability (SVV), and Total Fluid Content (TFC), which is actually the one over the BC measured impedance.**

**
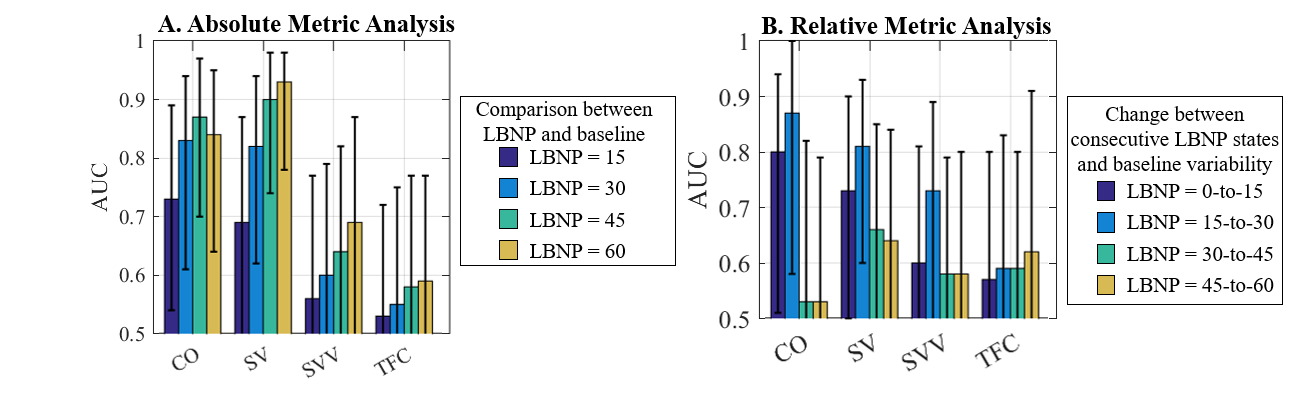
**

**Figure A.6. AUC of absolute (A) and relative (B) metrics output from the** b**ioimpedance cardiography device. Bars represent the AUC values and whiskers are the 95% confidence intervals.**

**A.5. EIT impedance subset**

We utilized a subset of impedance patterns when calculating the EIT_thx_ or EIT_ab_ metric. The IIVVs of the subset are defined in Table A.1 assuming each thorax and abdomen belt had electrodes labeled 1-16, with electrode 1 at the midline towards patients left and electrode 2 towards the patient’s front. IIVV’s are a way to describe four-electrode impedance measurements (patterns), which list the current driving electrodes as II and the voltage-measurement electrodes as VV.

**Table A.1. Subset of IIVV patterns.**

| **IIVV (1-18)** |  | **IIVV (19-36)** |  | **IIVV (37-50)** |
| --- | --- | --- | --- | --- |
| 6 1 3 8  6 1 5 10  6 1 8 13  6 1 9 14  6 1 10 15  7 2 4 9  7 2 6 11  7 2 9 14  7 2 10 15  7 2 11 16  8 3 1 6  8 3 5 10  8 3 7 12  8 3 10 15  8 3 11 16  9 4 2 7  9 4 6 11  9 4 8 13 |  | 9 4 11 16  10 5 1 6  10 5 3 8  10 5 7 12  10 5 9 14  11 6 2 7  11 6 4 9  11 6 8 13  11 6 10 15  12 7 3 8  12 7 5 10  12 7 9 14  12 7 11 16  13 8 1 6  13 8 4 9  13 8 6 11  13 8 10 15  14 9 1 6 |  | 14 9 2 7  14 9 5 10  14 9 7 12  14 9 11 16  15 10 1 6  15 10 2 7  15 10 3 8  15 10 6 11  15 10 8 13  16 11 2 7  16 11 3 8  16 11 4 9  16 11 7 12  16 11 9 14 |

**A.6. Details of final Random Forest Model Features**

The number of features used for each technology along with the list of feature names are provided in Table A.2.

**Table A.2. Number and features used to train the random forest models for each technology.**

| Technology | Number of Features | Feature Names |
| --- | --- | --- |
| MAP | 2 | Brachial Arterial, Mean Brachial Arterial |
| HR | 2 | ECG, HR |
| HRV | 20 | nni_mean, nni_min, nni_max, hr_mean, hr_min, hr_max, hr_std, sdnn, rmssd, sdsd, fft_ratio, fft_total, lomb_ratio, lomb_total, ar_ratio, ar_total, sd1, sd2, sd_ratio, ellipse_area |
| Pleth | 1 | Pleth |
| EIT_ab | 98 | EIT_01_ab, EIT_02_ab, … , EIT_98_ab |
| EIT_thx | 98 | EIT_01_thx, EIT_02_thx, … , EIT_98_thx |
| EIS_thx | 200 | EIS_X_thx_100Hz, EIS_X_thx_109Hz, … , EIS_X_thx_1000000Hz, EIS_R_thx_100Hz, … , EIS_R_thx_1000000Hz |
| EIS_ab | 200 | EIS_X_ab_100Hz, EIS_X_ab_109Hz, … , EIS_X_ab_1000000Hz, EIS_R_ab_100Hz, … , EIS_R_ab_1000000Hz |
| EIS_arm | 200 | EIS_X_arm_100Hz, EIS_X_arm_109Hz, … , EIS_X_arm_1000000Hz, EIS_R_arm_100Hz, … , EIS_R_arm_1000000Hz |
| HRV variables are described in A.7; EIT_[XX]_ab or EIT_[XX]_thx refers to the impedance pattern number (there were 98 patterns); in the EIS metrics, X refers to reactance and R refers to resistance. | | |

**A.7. Importance of Random Forest Models.**

This section provides a listing of the MDI (mean decrease in impurity) feature importance for the 20 most important random forest model features considered. Features are separated into vital signs (Fig. A.7), EIT features (Fig. A.8), and EIS features (Fig. A.9).


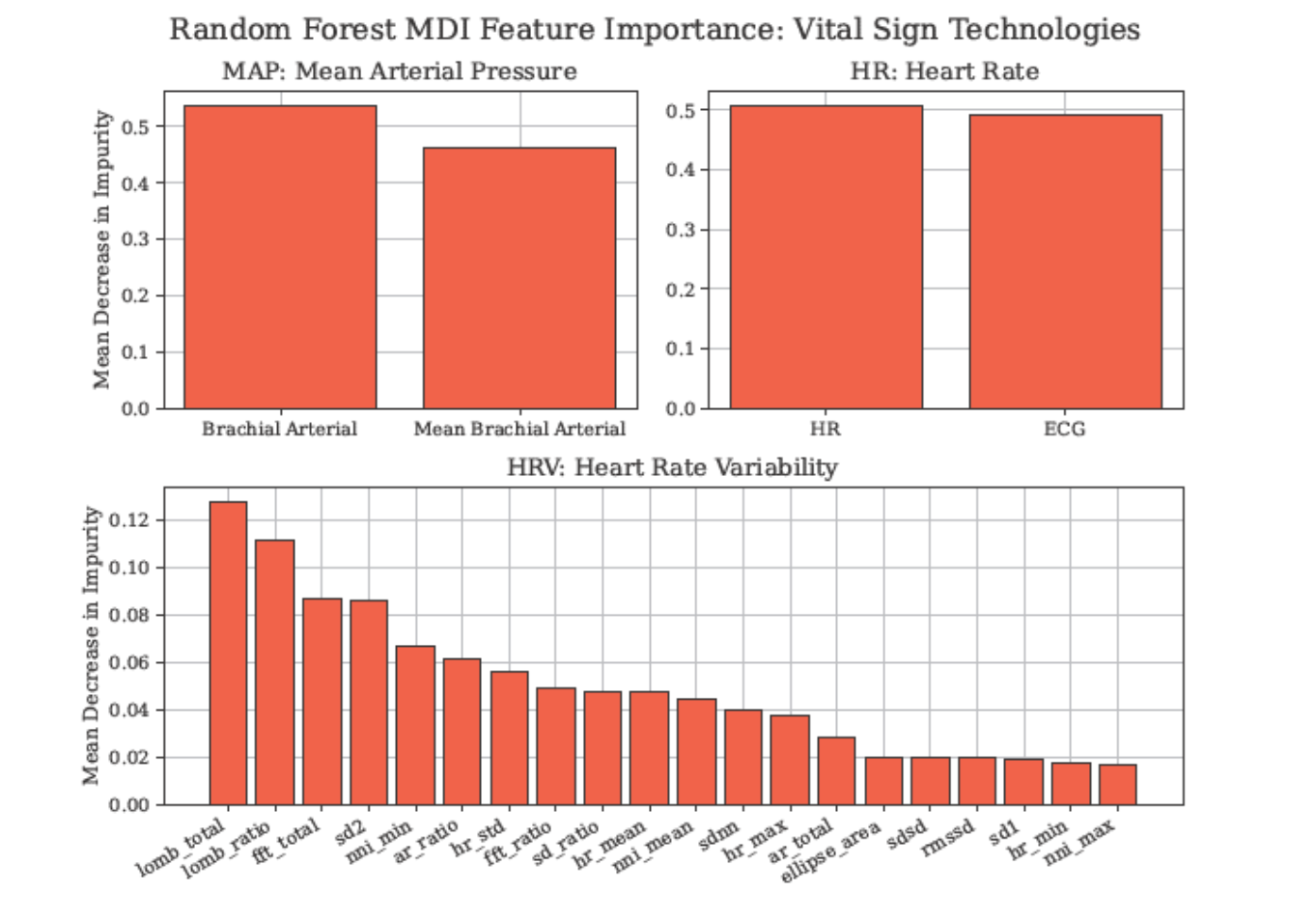


**Figure A.7. MDI feature importance for vital sign technologies.**

**
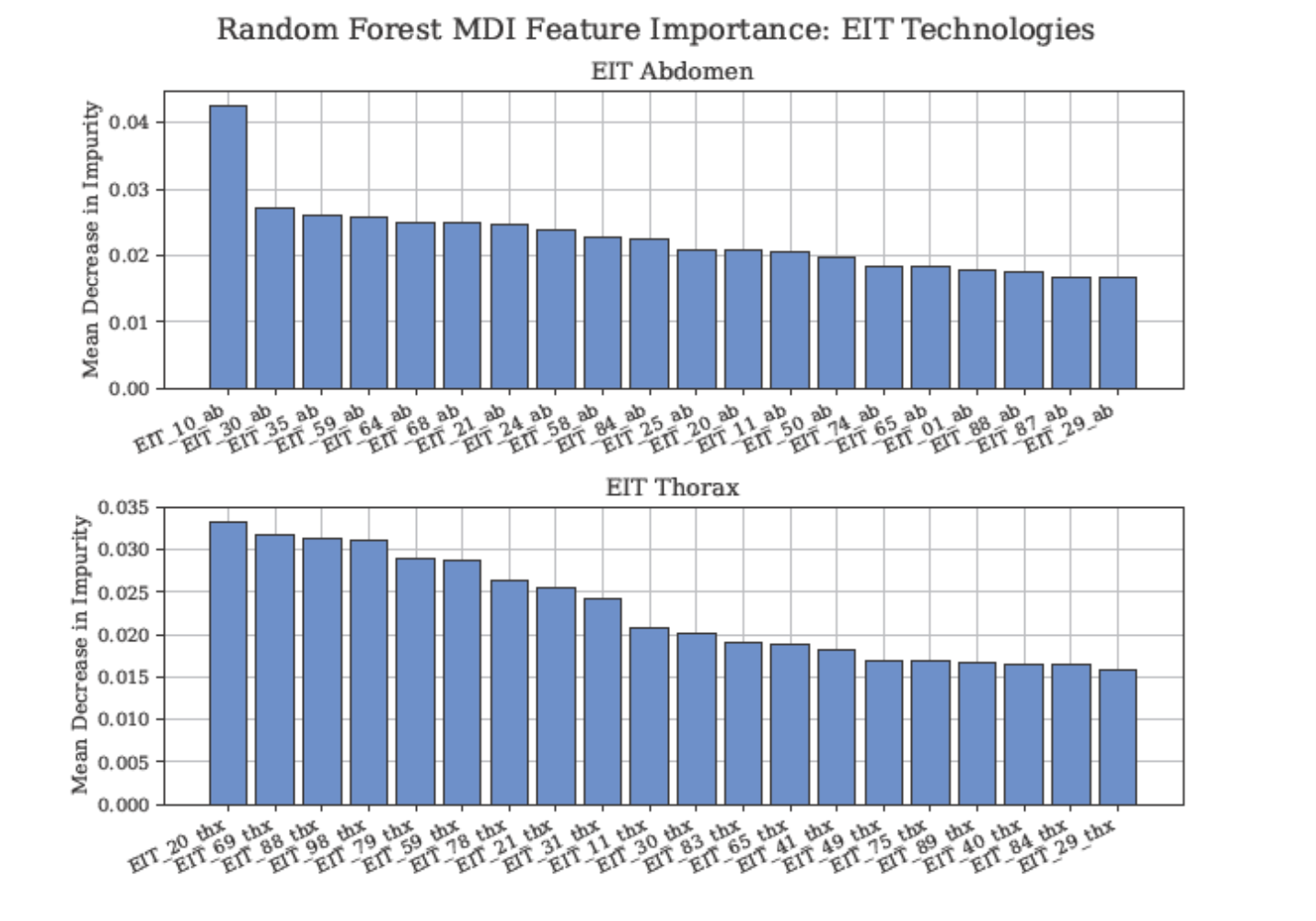
Figure A.8. MDI feature importance for EIT technologies.**


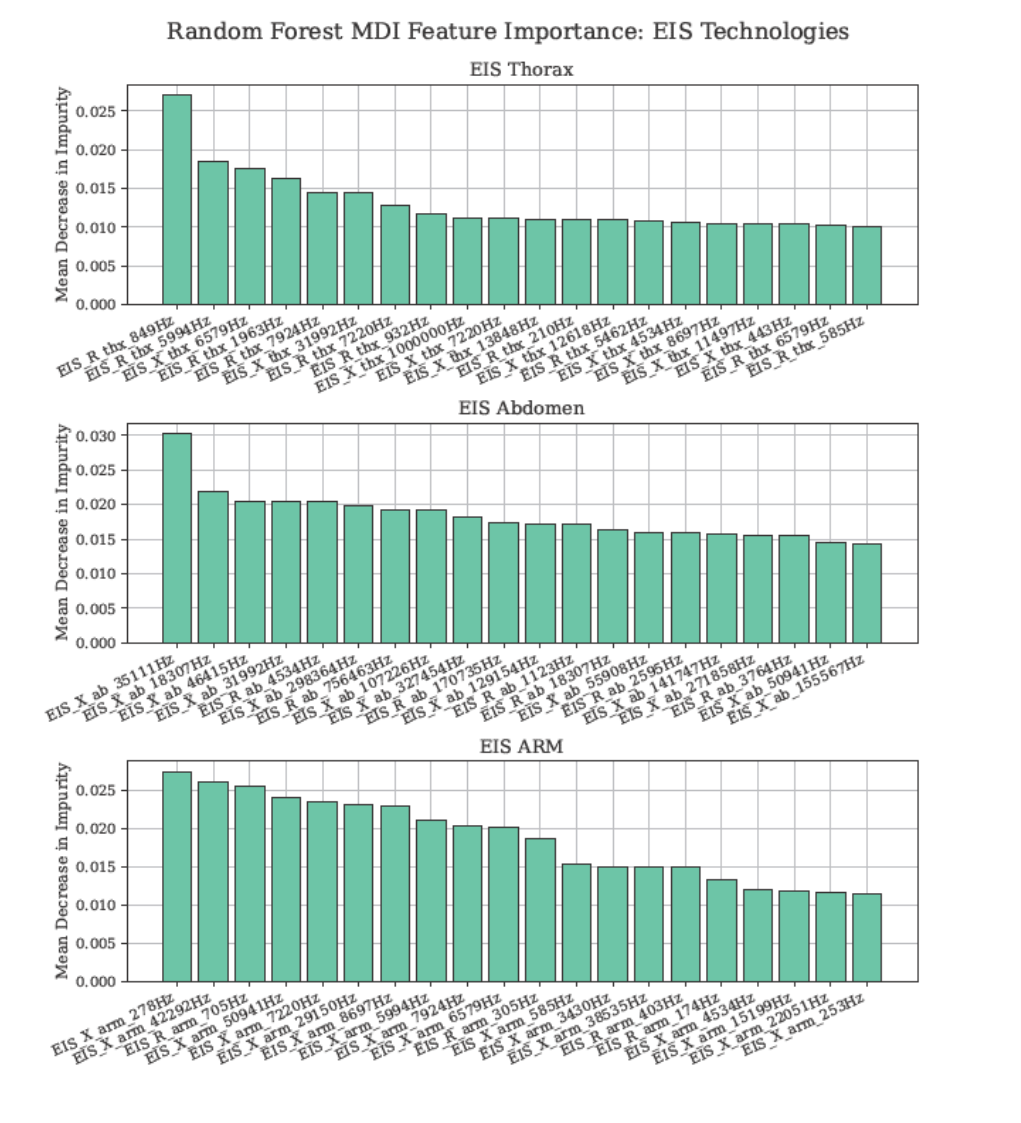


**Figure A.9. MDI feature importance for EIS technologies. Note that R represents resistance and X represents reactance in feature names.**

**A.8. PyHRV Library Features Extracted from Millisecond-level ECG Data**

PyHRV, a python library for heart rate variability, was used to generate 20 features for use in supervised learning methods from the time series ECG signals produced during the study derived from 9 metrics. The features generated fall into three groups: HRV time domain parameters, frequency domain parameters, and nonlinear parameters. The time domain parameters chosen (Table. A.3. 1-5) were generated by calculating basic statistics from NN (normal-to-normal) intervals and heart rate data. The frequency domain parameters chosen (Table. A.3. 6-8) were generated using Welch’s method, the Lomb-Scargle Periodogram, and autoregressive statistical methods. The nonlinear parameters chosen (Table. A.3. 9) were extracted from a Poincaré plot of a series of NN intervals and included information regarding the Poincaré ellipse.

**Table A.3. Table describing each metric number, metric name, description, and the features extracted from each metric using the PyHRV library. Descriptions are borrowed from PyHRV documentation [29].**

| **Metric Number** | **Metric Name** | **Description** | **Features Extracted** |
| --- | --- | --- | --- |
| 1 | nni_parameters | Computes basic statistical parameters from a series of NN intervals (# of intervals, mean, min, max) | nni_min, nni_max, nni_mean |
| 2 | hr_parameters | Computes basic statistical parameters from a series of heart rate (HR) data (mean, min, max, standard deviation) | hr_mean, hr_min, hr_max, hr_std |
| 3 | sdnn | Computes the Standard Deviation of a NN interval series (SDNN) | sdnn |
| 4 | rmssd | Computes the root mean of squared NN interval (NNI) differences. | rmssd |
| 5 | sdsd | Standard deviation of NNI differences. | sdsd |
| 6 | welch_psd | Computes a Power Spectral Density (PSD) estimation from the NNI series using the Welch’s method and computes all frequency domain parameters from this PSD according to the specified frequency bands. | fft_ratio, fft_total |
| 7 | lomb_psd | Computes a Power Spectral Density (PSD) estimation from the NNI series using the Lomb-Scargle Periodogram and computes all frequency domain parameters from this PSD according to the specified frequency bands. | lomb_ratio, lomb_total |
| 8 | ar_psd | Computes a Power Spectral Density (PSD) estimation from the NNI series using the autoregressive method and computes all frequency domain parameters from this PSD according to the specified frequency bands. | ar_ratio, ar_total |
| 9 | poincare | Creates the Poincaré plot from a series of NN intervals or R-peak locations and derives the Poincaré related parameters SD1, SD2, SD2/SD1 ratio, and area of the Poincaré ellipse. | sd1, sd2, sd_ratio, ellipse_area |
